# Household fuel use and migraine among Chinese adults aged 45 years and older: The modifying effects of sleep

**DOI:** 10.1371/journal.pone.0335407

**Published:** 2025-12-01

**Authors:** Jiamin Yan, Xiaomei Wang, Bangxian Ding, Minzhe Zhang, Qiqiang He, Ye Zeng, Yun Xiang

**Affiliations:** 1 Department of Laboratory Medicine, Wuhan Children’s Hospital (Wuhan Maternal and Child Healthcare Hospital), Tongji Medical College, Huazhong University of Science & Technology, Wuhan, P. R. China; 2 School of Public Health, Wuhan University, Wuhan, China; 3 Hubei Biomass-Resource Chemistry and Environmental Biotechnology Key Laboratory, Wuhan University, Wuhan, China; MeU: Mattu University, ETHIOPIA

## Abstract

**Background:**

Various studies have established a link between household fuel use and diverse health conditions. However, research examining the impact of household fuel use on migraine remains scarce. Therefore, the objective of this study was to explore the association between household fuel use and the risk of migraine, as well as the potential modifying effects of sleep duration.

**Methods:**

Utilizing a nationwide representative cohort from the China Health and Retirement Longitudinal Study (CHARLS) from 2011 to 2015, we included 9,160 participants aged 45 years and older who did not have migraine at baseline. Household fuel use was categorized into two groups: clean fuel and solid fuel. Migraine was defined based on self-reports using the ID-Migraine questionnaire. Sleep duration was classified into two groups: Non-ideal sleep duration (<7 hours/d or >8 hours/d) and ideal sleep duration (7–8 hours/d). Cox proportional hazards regression models were utilized to assess the associations of solid fuel use and sleep duration with migraine. The modifying effect of sleep duration was analyzed.

**Result:**

During a 4-year follow-up period, 520 migraine cases were identified. The use of solid fuels was associated with an increased risk of migraine compared to the use of clean fuels. The hazard ratio (HR) and 95% confidence interval (CI) was 1.34 (1.06–1.69) for heating and 1.29 (1.06–1.58) for cooking with solid fuels, compared to the use of clean fuels. The use of solid fuels for heating and cooking simultaneously was also associated with an elevated risk of migraine (HR: 1.53, 95% CI: 1.16–2.01), compared with the simultaneous use of clean fuels. Additionally, compared with consistent solid fuels users, those switching from solid to clean fuel and consistently using clean fuels for heating and cooking showed a decreased risk of migraine. For heating, the HR was 0.66 (95% CI: 0.49–0.90) for switching from solid to clean fuel and 0.54 (95% CI: 0.38–0.77) for consistently using clean fuels; for cooking, the corresponding HRs were 0.74 (95% CI: 0.57–0.97) and 0.68 (95% CI: 0.53–0.86), respectively. Ideal sleep duration modified the association between solid fuel use and migraine. Among individuals with non-ideal sleep duration, the use of solid fuels for heating (HR: 1.47, 95% CI: 1.09–1.97, P = 0.010) and cooking (HR: 1.31, 95% CI: 1.02–1.68, P value = 0.034) was significantly associated with an increased risk of migraine. In contrast, these associations were not statistically significant among those with ideal sleep duration (heating: HR: 1.13, 95% CI: 0.77–1.65, P = 0.537; cooking: HR: 1.28, 95% CI: 0.90–1.81, P = 0.165).

**Conclusion:**

Household solid fuel use was associated with an increased risk of migraine, and this association was modified by ideal sleep duration. Reducing exposure to household air pollution from solid fuel use and promoting healthy sleep behaviors may help to reduce the burden of migraine in areas where solid fuel use is prevalent.

## Introduction

Migraine is a common neurological disorder characterized by recurrent headaches that often lead to significant disability and a reduced quality of life. Globally, migraine affects approximately 14–15% of the population, translating to over 1 billion individuals, making it the second leading cause of years of life lived with disability among all causes of diseases [1–3]. In China, the 1-year prevalence of migraine was estimated as 9.3%, posing a major public health challenge [4]. Given the high prevalence of migraine and its substantial impact on quality of life and productivity, there is an urgent need to identify risk factors for migraine in order to initiate public health interventions to reduce the burden of migraine.

Environmental factors such as household air pollution (HAP) have become research focus due to their significant impact on health [5]. Solid fuels such as wood, coal and crop residues are widely used for heating and cooking, particularly in low- and middle-income countries (LMICs) [6]. In China, the government has implemented large-scale interventions to promote a transition from coal and biomass to clean energy sources, such as natural gas and electricity. For example, the “Air Pollution Prevention and Control Action Plan” launched in 2013, has contributed to notable progress in reducing solid fuel use [7]. Between 2014–2015 and 2019–2020, solid fuel use showed a marked decline, with cooking fuel use falling from 45.3% to 28.0% and heating fuel use decreasing from 33.5% to 23.2% [8]. However, the transition remains incomplete, and the health impacts of both historical and current solid fuel use may continue to pose major concerns. Previous studies have linked HAP from solid fuel combustion with a range of health outcomes, including chronic obstructive pulmonary disease (COPD), cardiovascular disease, impaired cognitive function, and headache [9–12]. However, while headache has been related to HAP exposure, migraine is a distinct headache disorder with unique pathophysiology and significant global burden, and it remains largely underexplored [13,14].

Epidemiologic studies have documented an association between sleep disturbances and migraine. Sleep disturbance has consistently been identified as a strong trigger for migraine attacks, with poor sleep quality and inadequate sleep duration as risk factors for migraine occurrence and severity [15–17]. Beyond this link, accumulating evidence suggests that sleep may also play a modifying role in the relation between environmental hazards and health. For instance, a recent study of the UK Biobank cohort showed that a healthy sleep pattern could mitigate the adverse effects of particulate matter 2.5 (PM_2.5_) exposure on dementia risk [18]. Similarly, another study found that better sleep quality attenuated accelerated biological aging induced by air pollution [19]. However, no study to date has examined the modifying effect of sleep on the relationship between household fuel use and migraine.

Therefore, the aim of this study was to investigate the association between household solid fuel use and migraine risk, as well as the potential modifying role of sleep. We hypothesized that individuals with household solid fuel use would have an increased risk of migraine, and this association was attenuated by ideal sleep duration.

## Methods

### Study population

**Fig 1:**
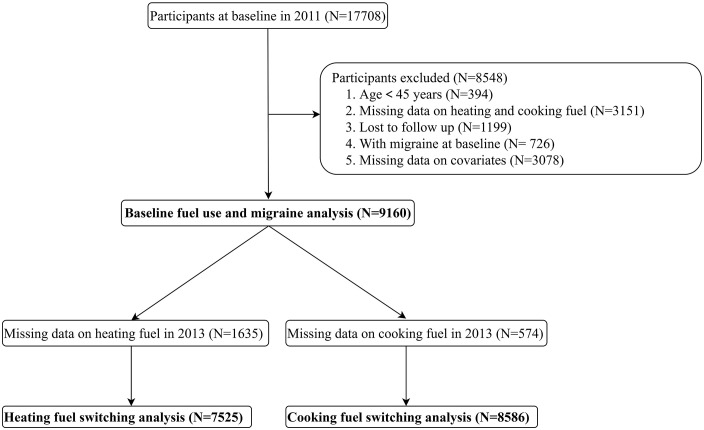
Selection flow chart of study participants.

The data was used from the China Health and Retirement Longitudinal Study (CHARLS), a nationally representative longitudinal survey conducted by Peking University. CHARLS collected extensive data on various domains, including demographics, health, economics, social networks and support, health behaviors, and health insurance and medical case on individuals aged 45 years and older. Employing a multistage and stratified probability sampling method, CHARLS covered over 17,000 individuals from approximately 10,000 households across 150 counties/districts and 450 villages/urban communities. It was launched in 2011 and there were five survey waves so far (2011, 2013, 2015, 2018 and 2020). In this study, we used data from 2011, 2013 and 2015, as the use of heating fuels was only available in 2011 and 2013. Participants aged <45 years, and those who had no data on household fuel use, lost to follow-up, with migraine in 2011 and missing values on covariates were excluded, leaving 9,160 participants for the analysis of household fuel use and migraine. For the analysis of fuel switching between 2011 and 2013, participants were divided into four groups: consistent use of clean fuels, switching from solid to clean fuel, switching from clean to solid fuel, and consistent use of solid fuels. The analysis of heating fuel switching included 7,525 participants after excluding 1,635 individuals with missing data in 2013. Similarly, the analysis of cooking fuel switching involved 8,586 participants after excluding 574 individuals with missing cooking information on 2013 (**Fig 1**).

### Migraine

Migraine is a primary headache disorder typically characterized by recurrent, moderate-to-severe headaches often associated with symptoms like nausea, photophobia, and phonophobia [20]. In this study, migraine cases were identified from 2013 to 2015 using the three-item ID-Migraine questionnaire, a validated screening tool with a sensitivity of 0.81 (95% CI, 0.77–0.85) and a specificity of 0.75 (95% CI, 0.64–0.84) for migraine detection in clinical and population-based settings [21]. The three screening questions were: (1) Have you suffered from nausea or stomach discomfort? (2) Have your headaches prevented you from carrying out your daily tasks? (3) Are you much more sensitive to light when you do not have a headache? A diagnosis of migraine was made if two or more of these criteria were met. In the CHARLS dataset, responses to “currently feel stomach pain” and “currently feel headache” correspond to the first and second items of the ID-Migraine questionnaire. Therefore, individuals reporting both symptoms were classified as having migraine [22,23].

### Household fuel use

Household fuel use was assessed by asking participants two key questions: “What is the primary energy source for heating?” and “What is the main fuel used for cooking?”. The response options for heating fuel were: solar, coal, natural gas, liquefied petroleum gas, electricity, crop residue/wood burning, and other. For cooking fuel, the options were: coal, natural gas, marsh gas, liquefied petroleum gas, electricity, crop residue/wood burning, uniform heating, and other. Solid fuels, such as coal and crop residue/wood burning, were classified based on their high emission of indoor air pollutants, including PM_2.5_, carbon monoxide (CO), and nitrogen dioxide (NO_2_). Clean fuel, such as natural gas, liquefied petroleum gas, electricity, and solar energy, produced lower levels of these pollutants. This classification aligns with guidelines from the World Health Organization on household air pollution [24]. For this study, solid fuel for heating and cooking was defined as coal and crop residue/wood burning. Clean fuel for heating was defined as solar, natural gas, liquefied petroleum gas, and electricity, while clean fuel for cooking was defined as natural gas, marsh gas, liquefied petroleum gas, electricity, and uniform heating. The response of “other” was excluded to avoid misclassification.

### Sleep duration

Sleep duration was assessed at baseline in 2011 by asking “During the past month, how many hours of actual sleep did you get at night (average hours for one night)?” Previous study has shown that 7–8 hours/d of sleep was most beneficial for overall health outcomes. In line with this, the National Sleep Foundation recommends this sleep duration for older adults, deeming it ideal for supporting their general well-being [25,26]. Thus, we categorized sleep duration into ideal sleep duration (7–8 hours/d) and non-ideal sleep duration (<7 hours/d or >8 hours/d).

### Covariates

Covariates included in the analysis were selected based on previous study linking demographic, socioeconomic, and health-related factors to migraine [22,27,28]. These variables included age (<65 years, ≥ 65 years), sex (male, female), education level (primary school or below, middle school or above), marital status (married, single or other), residence (rural, urban), smoking status (yes, no), and alcohol consumption (yes, no). Additionally, body mass index (BMI) was calculated as weight in kilograms divided by height in meters squared. Comorbid conditions such as hypertension, dyslipidemia, and diabetes were also included as covariates defined by self-reported physician diagnoses or the use of relevant medication. Household income was categorized into low and high based on median income distribution.

### Statistical analysis

Descriptive statistics were conducted to summarize the baseline characteristics of the study population based on heating and cooking fuel use. The normality of continuous data was assessed using the Kolmogorov-Smirnov test. Given the non-normal distribution of age and BMI, these variables were expressed as medians with interquartile ranges (IQRs). Categorical variables were presented as frequencies and percentages. Comparisons between groups were performed using the chi-square test for categorical variables and the Mann-Whitney U test for continuous variables.

Cox proportional hazards regression models were used to estimate the hazard ratio (HR) and 95% confidence interval (CI) for the association between household fuel use, sleep duration and migraine risk. The time-to-event variable was defined as the duration from the baseline survey to the onset of migraine or to the end of follow-up. A stepwise approach was used for adjustment: an unadjusted model and a fully adjusted model. The adjusted model controlled for variables such as age, sex, education level, marital status, residence, smoking status, alcohol consumption, sleep duration, household income, BMI, and comorbid conditions (hypertension, dyslipidemia, and diabetes). Multicollinearity among covariates was assessed using variance inflation factors (VIF) with the R package “car”. All VIF values were below 5, indicating no significant collinearity among the variables. For the analysis of fuel switching between 2011 and 2013, cox regression models were used to examine the risk of migraine in these categories, with consistent use of clean fuels serving as the reference group. Subgroup analyzes were conducted to further investigate the association between household fuel use and migraine risk using covariates such as age, sex, education level, residence, smoking status, alcohol consumption, hypertension, and household income. Potential effect modification was assessed by adding interaction terms to the Cox proportional hazards models, estimating HR and 95% CI for each subgroup. Interaction P values were determined by likelihood ratio tests. We also examined the proportional hazards assumption using Schoenfeld residuals, and no significant violations were detected.

All statistical analyses were conducted using R software (version 4.3.2). The proportional hazards assumption for the Cox models was evaluated using Schoenfeld residuals, and no violations were identified. Statistical significance was set at a two-sided P value of less than 0.05.

### Ethics approval and consent to participate

The data used in this study were approved by the Biomedical Ethics Review Committee of Peking University (Approval Number: IRB00001052–11015). Written informed consent was obtained from all participants. All methods in this study on humans described in the manuscript were performed in accordance with national law and the Helsinki Declaration of 1975. Clinical trial number: not applicable.

## Results

### Baseline characteristics of study participants

**Table 1** shows the baseline characteristics of the study participants based on their fuel use for heating and cooking in the household. A total of 9,160 participants were included in the study, of which 6,244 (68.2%) used solid fuel for heating and 5,216 (56.9%) used solid fuel for cooking. Participants who used solid fuel for heating were older (57.00 years (50.00, 64.00) vs 59.00 years (52.00, 65.00), P value: < 0.001), had a lower BMI (22.86 kg/m^2^ (20.61, 25.59) vs. 23.95 kg/m^2^ (21.71, 26.48), P value: < 0.001), were more likely to live in rural areas (74.8% vs. 36.0%, P value: < 0.001), had a lower household income (60.5% vs. 31.2%, P value: < 0.001), tended to be smoker (42.5% vs. 37.1%, P value: < 0.001), and had a lower prevalence of hypertension (22.7% vs. 25.7%, P value: < 0.001), dyslipidemia (8.1% vs. 11.7%, P value: < 0.001) and diabetes (4.8% vs. 7.4%, P value: < 0.001), compared to those who used clean fuels. A similar trend was observed among participants who used solid fuel for cooking. The proportion of participants with non-ideal sleep duration was higher among solid fuel users for heating (58.1% vs. 54.7%, P value: 0.002) and cooking (58.7% vs. 54.8%, P value: < 0.001) than clean fuel users.

**Table 1:** Characteristics of study participants according to household fuel use.

Variable		Heating			Cooking		
	All participants (N = 9160)	Clean fuel (N = 2916)	Solid fuel (N = 6244)	P value	Clean fuel (N = 3944)	Solid fuel (N = 5216)	P value
^1^Age (years)	59.08 (9.29)	57.00 (50.00, 64.00)	59.00 (52.00, 65.00)	<0.001	57.00 (50.00, 64.00)	59.00 (53.00, 66.00)	<0.001
^1^BMI (kg/m^2^)	23.64 (4.01)	23.95 (21.71, 26.48)	22.86 (20.61, 25.59)	<0.001	23.87 (21.49, 26.45)	22.75 (20.55, 25.42)	<0.001
^2^Sex, n (%)				0.124			0.179
Male	4,460 (48.7)	1,385 (47.5)	3,075 (49.2)		1,888 (47.9)	2,572 (49.3)	
Female	4,700 (51.3)	1,531 (52.5)	3,169 (50.8)		2,056 (52.1)	2,644 (50.7)	
^2^Education level, n (%)				<0.001			<0.001
Primary school or below	6,060 (66.2)	1,506 (51.6)	4,554 (72.9)		2,131 (54.0)	3,929 (75.3)	
Middle school or above	3,100 (33.8)	1,410 (48.4)	1,690 (27.1)		1,813 (46.0)	1,287 (24.7)	
^2^Marital status, n (%)				0.998			0.907
Married	8,084 (88.3)	2,574 (88.3)	5,510 (88.2)		3,483 (88.3)	4,601 (88.2)	
Single or other	1,076 (11.7)	342 (11.7)	734 (11.8)		461 (11.7)	615 (11.8)	
^2^Residence, n (%)				<0.001			<0.001
Rural	5,719 (62.4)	1,051 (36.0)	4,668 (74.8)		1,621 (41.1)	4,098 (78.6)	
Urban	3,441 (37.6)	1,865 (64.0)	1,576 (25.2)		2,323 (58.9)	1,118 (21.4)	
^2^Smoking status, n (%)	3,733 (40.8)	1,082 (37.1)	2,651 (42.5)	<0.001	1,509 (38.3)	2,224 (42.6)	<0.001
Alcohol consumption, n (%)	3,109 (33.9)	1,004 (34.4)	2,105 (33.7)	0.514	1,364 (34.6)	1,745 (33.5)	0.268
^2^Household income, n (%)				<0.001			<0.001
Low	4,686 (51.2)	910 (31.2)	3,776 (60.5)		1,452 (36.8)	3,234 (62.0)	
High	4,474 (48.8)	2,006 (68.8)	2,468 (39.5)		2,492 (63.2)	1,982 (38.0)	
^2^Hypertension, n (%)	2,165 (23.6)	748 (25.7)	1,417 (22.7)	0.002	998 (25.3)	1,167 (22.4)	0.001
^2^Dyslipidemia, n (%)	844 (9.2)	341 (11.7)	503 (8.1)	<0.001	442 (11.2)	402 (7.7)	<0.001
^2^Diabetes, n (%)	515 (5.6)	217 (7.4)	298 (4.8)	<0.001	278 (7.0)	237 (4.5)	<0.001
^2^Sleep duration, n (%)				0.002			<0.001
Non-ideal sleep duration	5,223 (57.0)	1,594 (54.7)	3,629 (58.1)		2,163 (54.8)	3,060 (58.7)	
Ideal sleep duration	3,937 (43.0)	1,322 (45.3)	2,615 (41.9)		1,781 (45.2)	2,156 (41.3)	

^1^Mann-Whitney U Test; ^2^Pearson’s Chi-squared test.

### Association of household fuel use and sleep duration with migraine risk

**Table 2** shows the association of solid fuel use and sleep duration with migraine risk. In the unadjusted model, the use of solid fuel for heating (HR: 1.88, 95% CI: 1.51–2.32) and for cooking (HR: 1.74, 95% CI: 1.45–2.10) were both associated with a higher risk of migraine. Participants who used solid fuels for both heating and cooking had the highest risk (HR: 2.28, 95% CI: 1.78–2.93). After adjustment for potential confounders, these associations remained statistically significant, although the effect estimates were attenuated (heating: HR: 1.34, 95% CI: 1.06–1.69; cooking: HR: 1.29, 95% CI: 1.06–1.58; both heating and cooking: HR: 1.53, 95% CI: 1.16–2.01). Regarding sleep, participants with ideal sleep duration had a significantly lower risk of migraine, both in the unadjusted (HR: 0.67, 95% CI: 0.55–0.80) and adjusted models (HR: 0.73, 95% CI: 0.61–0.88).

**Table 2:** Association of household fuel use and sleep duration with migraine risk.

Variable	Number of cases/Total	Unadjusted model		Adjusted model	
		HR (95% CI)	P value	HR (95% CI)	P value
Household fuel use					
Heating fuel					
Clean fuel	105/2916	Reference		Reference	
Solid fuel	415/6244	1.88 (1.51-2.32)	<0.001	1.34 (1.06-1.69)	0.013
Cooking fuel					
Clean fuel	159/3944	Reference		Reference	
Solid fuel	361/5216	1.74 (1.45-2.10)	<0.001	1.29 (1.06-1.58)	0.012
Heating and cooking					
Clean fuel use	75/2360	Reference		Reference	
Mixed fuel use	114/2140	1.69 (1.27-2.27)	<0.001	1.33 (0.98-1.79)	0.066
Solid fuel use	331/4660	2.28 (1.78-2.93)	<0.001	1.53 (1.16-2.01)	0.003
Sleep duration					
Non-ideal sleep duration	345/5223	Reference		Reference	
Ideal sleep duration	175/3937	0.67 (0.55-0.80)	<0.001	0.73 (0.61-0.88)	0.001

Adjusted for age, sex, education level, marital status, residence, smoking status, alcohol consumption, household income, hypertension, dyslipidemia, diabetes, BMI.

### Association between fuel switching patterns and migraine risk

**Table 3** illustrates the association between fuel switching types and migraine risk. In the adjusted model, switching from solid to clean fuel (HR: 0.66, 95% CI: 0.49–0.90) and consistently using clean fuels (HR: 0.54, 95% CI: 0.38–0.77) for heating were significantly associated with a decreased risk of migraine compared to consistently using of solid fuels. Similarly, switching from solid to clean fuel (HR: 0.74, 95% CI: 0.57–0.97) and consistently using clean fuels (HR: 0.68, 95% CI: 0.53–0.86) for cooking were linked to a lower risk of migraine in comparison to those who consistently used solid fuels.

**Table 3:** Association between household fuel use switching and migraine risk.

Variable	Number of cases/Total	Unadjusted model		Adjusted model	
		HR (95% CI)	P value	HR (95% CI)	P value
Heating fuel switching types					
Both solid fuels	317/4307	Reference		Reference	
Clean to solid fuel	30/560	0.72 (0.49-1.05)	0.085	0.84 (0.58-1.23)	0.374
Solid to clean fuel	49/1085	0.60 (0.45-0.81)	0.001	0.66 (0.49-0.90)	0.008
Both clean fuels	42/1573	0.35 (0.26-0.49)	<0.001	0.54 (0.38-0.77)	0.001
Cooking fuel switching types					
Both solid fuels	279/3650	Reference		Reference	
Clean to solid fuel	39/618	0.82 (0.59-1.15)	0.244	0.90 (0.64-1.26)	0.538
Solid to clean fuel	68/1299	0.68 (0.52-0.88)	0.004	0.74 (0.57-0.97)	0.029
Both clean fuels	113/3019	0.48 (0.39-0.60)	<0.001	0.68 (0.53-0.86)	0.002

Adjusted for age, sex, education level, marital status, residence, smoking status, alcohol consumption, household income, hypertension, dyslipidemia, diabetes, BMI.

### Effect modification by sleep duration

**Table 4** shows the modifying effect by sleep duration on the association between household fuel use and migraine risk. Among participants with non-ideal sleep duration, the use of solid fuel for heating was significantly associated with a higher risk of migraine (HR: 1.47, 95% CI: 1.09–1.97, P = 0.010), while no significant association was observed in participants with ideal sleep duration (HR: 1.13, 95% CI: 0.77–1.65, P = 0.537). A similar pattern was found for cooking fuel, with a significant association in those with non-ideal sleep duration (HR: 1.31, 95% CI: 1.02–1.68, P = 0.034), but not those with ideal sleep duration (HR: 1.28, 95% CI: 0.90–1.81, P = 0.165).

**Table 4:** Association between household fuel use and migraine risk according to sleep duration.

Variable	Non-ideal sleep duration		Ideal sleep duration	
	HR (95% CI)	P value	HR (95%CI)	P value
Heating fuel				
Clean fuel	Reference		Reference	
Solid fuel	1.47 (1.09-1.97)	0.010	1.13 (0.77-1.65)	0.537
Cooking fuel				
Clean fuel	Reference		Reference	
Solid fuel	1.31 (1.02-1.68)	0.034	1.28 (0.90-1.81)	0.165

Adjusted for age, sex, education level, marital status, residence, smoking status, alcohol consumption, household income, hypertension, dyslipidemia, diabetes, BMI.

### Subgroup analyses

**Fig 2:**
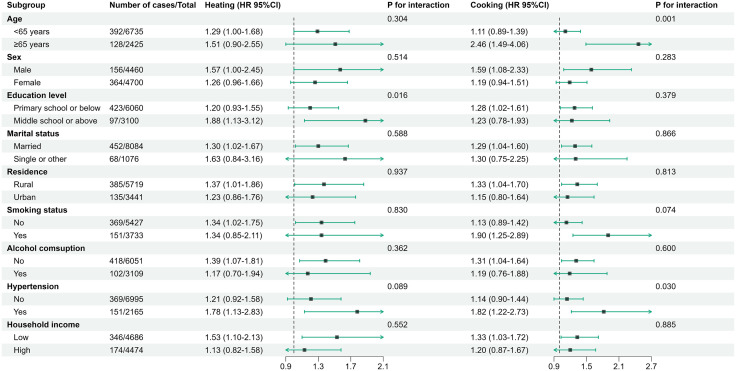
Subgroup analyses on the association of household fuel use with migraine risk.

**Fig 2** displays the results of subgroup analyses exploring the association between household fuel use and migraine risk. A stronger association between solid fuel use for heating and migraine risk was observed in participants with higher education levels. Similarly, a stronger association between solid fuel use for cooking and migraine risk was identified among participants aged over 65 years and those with hypertension.

## Discussion

In this nationally representative prospective cohort study, we found that the use of solid fuels for both heating and cooking was significantly associated with an increased risk of migraine in Chinese middle-aged and elderly adults. This association remained robust even after adjusting for potential confounders such as sociodemographic factors and lifestyle behaviors. In addition, we found that the association between solid fuel use and migraine risk was modified by sleep duration, with non-significant associations observed among participants with ideal sleep duration. Our findings suggest that while transitioning to clean fuels is a critical public health priority for primary prevention, maintaining an ideal sleep duration may also play a protective role by mitigating some of the adverse effects of unavoidable exposure to household air pollution.

Migraine is a specific and debilitating primary headache disorder characterized by distinct features, such as nausea and sensory sensitivities, which set it apart from tension-type headaches or other general headaches [29]. Our findings are consistent with previous research showing an association between household air pollution from solid fuel use and headache, particularly in low- and middle-income countries where solid fuel use is more prevalent. In a cross-sectional study conducted in rural India, an association was found between the use of biomass fuels for cooking and headache symptoms [30]. A randomized controlled trial in rural Guatemala found that participants who used a plancha stove suffered fewer headaches than those who used open fires in the home. The plancha stove was equipped with a metal chimney that directed the smoke outside, reducing biomass smoke pollution compared to open fires [31]. Findings from the CLEAN-Air (Africa) study in peri-urban areas of Cameroon, Ghana and Kenya showed that participants who cooked exclusively with liquefied petroleum gas (LPG) were less likely to suffer from headaches than participants who used a combination of LPG and solid fuels such as charcoal and wood [32]. While these studies focused on general headache symptoms, our study provides the first evidence specifically for migraine, a more severe and well-defined neurological disorder. These studies on headache show a valuable foundation, as the underlying mechanisms, such as systemic inflammation and oxidative stress triggered by pollutants, are plausible pathways for triggering headaches in general. However, the link may be even stronger for migraine, as the pathophysiology of migraine involves a state of neuronal hyperexcitability and sensitization of the trigeminovascular system [33]. By identifying a robust association between solid fuel use and migraine, the present study adds to the existing knowledge and highlights the importance of migraine as a specific outcome in environmental health research.

The mechanisms linking solid fuel to migraine remain unclear, but insights can be drawn from the established relationship between outdoor air pollution and migraine. Household air pollution, primarily from the use of solid fuels such as wood, coal or crop residues, generates harmful pollutants such as PM, nitrogen dioxide (NO_2_), and carbon monoxide (CO). PM_2.5_ has been shown to induce the release of pro-inflammatory cytokines and reactive oxygen species, which can promote neuroinflammation and sensitization of the trigeminal nerve, a key player in migraine attacks [34]. Similarly, NO_2_ has been found to induce migraine in rats by activating the ROS-TRPV1 signaling pathway, which was associated with oxidative stress and inflammation [35]. CO exposure from incomplete combustion of solid fuel can lead to hypoxia, which has been implicated in the development of migraine [36]. In addition, the household environment, where ventilation is often poor, creates prolonged exposure to these harmful pollutants, amplifying the risk. Over time, chronic exposure to household air pollution could lead to persistent neuroinflammation, autonomic dysfunction, and a heightened state of neural excitability, increasing the migraine susceptibility.

Sleep is vital for overall health, playing a restorative role essential for both physical and mental well-being. Disorders related to sleep have been associated with a range of diseases, including cancer, cardiovascular disease, diabetes, depression, and migraine [37–39]. Cognitive Behavioral Therapy for Insomnia (CBT-I) is a structured approach designed to address unhelpful thoughts and behaviors related to sleep, ultimately improving sleep quality and tackling insomnia. Research has shown that CBT-I may have a beneficial effect on migraine symptoms [40,41]. One potential mechanism by which ideal sleep duration alleviates migraine risk is through the stabilization of cortical excitability, which is often heightened in individuals with migraine [42,43]. Sleep disturbances, such as poor sleep quality or irregular sleep patterns, may exacerbate cortical hyperexcitability, increasing susceptibility to migraine triggers, including environmental exposures like household air pollution from solid fuel use. In contrast, ideal sleep duration could reduce cortical responsiveness to these triggers, mitigating the risk of migraine onset. Another plausible mechanism involves the trigeminovascular system, a central pathway that plays a role in the pathophysiology of migraine [44,45]. Poor sleep has been shown to increase the sensitivity of the trigeminal nerve and increase susceptibility to pain stimuli and inflammatory responses triggered by air pollutants such as PM and NO_2_. In addition, sleep disturbances can impair the release of melatonin, a hormone with anti-inflammatory and antioxidant properties that regulates pain processing [46,47]. Reduced melatonin levels are associated with increased oxidative stress and neuroinflammation, both of which contribute to the development of migraines. Moreover, sleep is closely related to circadian rhythms, which influences inflammatory and oxidative stress pathways [48,49]. Exposure to pollutants from solid fuel may trigger inflammatory responses, leading to the development of migraine. Ideal sleep duration, by maintaining regular circadian rhythms, could mitigate these inflammatory responses, thus lowering migraine risk. The consistency of this pattern across both heating and cooking fuel use strengthens the evidence for sleep as an effect modifier. These findings suggest that ideal sleep duration could provide a protective buffer against the adverse neurological effects of household air pollution. In other words, individuals with non-ideal sleep duration may be particularly vulnerable to pollution-related migraine risk, whereas those maintaining ideal sleep duration appear more resilient to environmental triggers.

The subgroup analysis showed that people with a higher level of education were more likely to develop migraine when using solid fuels for heating, while people ≥65 years of age and with hypertension were more prone to migraine when using solid fuels for cooking. The increased risk in people with a higher level of education may be attributed to their increased social and life pressures, such as professional competition, work commitments and personal expectations. Chronic stress and anxiety, which are common in a high-pressure life, are known triggers for migraine [50]. Consistent with previous studies, older adults are more susceptible to indoor air pollution as the aging process gradually may weaken the immune system and reduce the ability to excrete inhaled pollutants, leading to increased systemic inflammation and oxidative stress that are central mechanisms in the development of migraine [51,52]. Furthermore, older adults may have longer cumulative exposure to household solid fuels. As CHARLS lacked precise measures of exposure duration, future studies incorporating detailed lifetime exposure histories are warranted. The increased susceptibility to migraines among individuals with hypertension may be attributed to the close association between hypertension and vascular changes, whereas migraine is thought to involve dysregulation of cerebral blood flow [53]. This aligns with previous studies showing that individuals with chronic conditions such as hypertension, diabetes, and cardiovascular disease are more vulnerable to the adverse effects of air pollution [54,55].

The results of this study have important public health implications, particularly for regions where the use of solid fuels remains widespread. Given the well-documented negative health impacts of household air pollution, switching to clean energy sources should be a priority for public health action. Policies to promote the use of clean fuels, such as subsidies for cleaner stoves and fuels or the introduction of indoor air quality standards, could play a critical role in reducing the risk of migraine and other pollution-related diseases. In addition, our study highlights the need for an integrated approach to migraine prevention that takes into account both environmental and lifestyle factors. Public health interventions targeting sleep health, in combination with efforts to reduce indoor air pollution, could provide a dual benefit in reducing migraine risk.

This study has several strengths, including its large sample size, community-based longitudinal design, and full adjustment for potential confounders. However, some limitations should be acknowledged. Firstly, the study only assessed fuel use at two time points (2011 and 2013). This might underestimate the long-term impacts of household fuel on migraine risk. Secondly, the study assessed sleep duration at a single time point. Since sleep duration may fluctuate due to various factors such as stress, illness, and environmental changes, multiple assessments of sleep behavior are warranted to reveal the interaction between sleep and household fuel use on migraine risk. Furthermore, our definition of ideal sleep duration (7–8 hours) was based on general population recommendations and did not account for individual variability in sleep needs, which may differ by age, sex, and other factors. Future studies with more refined assessments of ideal sleep duration that consider individual and demographic differences are warranted. Thirdly, the CHARLS dataset did not include ID-Migraine item three “Are you much more sensitive to light when you do not have a headache?”. The exclusion of the photophobia component may lower the sensitivity but not the specificity. Thus, the migraine diagnosis based on ID-Migraine item one and item two could lead to an underestimation of migraine cases and a conservative estimate of the association between solid fuel use and migraine. Fourthly, the reliance on self-reported migraine and sleep behavior may introduce reporting bias. Fifthly, although several factors have been controlled, some unmeasured confounders such as stress and diet, may have influenced the observed associations. Finally, the study only included middle aged and elderly Chinese, and this may limit the generalizability of the findings to other populations with different fuel use practices and environmental conditions.

## Conclusion

In conclusion, the use of solid fuels for heating and cooking was associated with an increased risk of migraine, and this association was attenuated in individuals with ideal sleep duration. Transitioning to clean fuel could have a positive impact on migraine, although further research is needed to confirm these results. Public health strategies that promote clean energy use and ideal sleep duration may reduce the burden of migraine.

### Contributions to the literature

- This study is the first to establish a longitudinal association between household solid fuel use and increased migraine risk in a nationally representative cohort of middle-aged and elderly Chinese adults, filling a crucial gap in environmental migraine research.
- The findings emphasize the public health importance of promoting the use of clean fuels to reduce the migraine burden in regions where solid fuel use is still widespread.
- Ideal sleep duration can mitigate the negative effects of household air pollution on migraine. Thus, healthy sleep pattern could act as a potential target for intervention.
